# Design Space Assessment of Virus Inactivation in Plasma‐Derived Matrices Establishes a Single Detergent Alternative to TX‐100‐Containing Mixes

**DOI:** 10.1002/bit.70232

**Published:** 2026-05-06

**Authors:** Johanna Kindermann, Cecilie Klausen, Michael Karbiener, Thomas R. Kreil

**Affiliations:** ^1^ Global Pathogen Safety Takeda Manufacturing Austria AG Vienna Austria

**Keywords:** biopharmaceuticals, ecofriendly detergent, plasma‐derived medicines, S/D treatment, viral safety, virus inactivation

## Abstract

The detergent Triton X‐100 (TX‐100) is a typical component of solvent/detergent (S/D) mixes applied for virus inactivation steps of biomanufacturing processes, yet environmentally unfavorable properties have led to restrictions on its use. Deviron 13‐S9 has recently been described as an eco‐friendly TX‐100 alternative, with powerful virus‐inactivating properties determined in matrices representative of recombinant and some plasma‐derived process intermediates such as cryo‐poor plasma. The present study extends this knowledge via a side‐by‐side comparison of virus inactivation by Deviron 13‐S9, applied as a single detergent, and a traditional TX‐100‐containing S/D mix, in four plasma‐derived matrices, bracketing a broad range of protein concentrations and pH values. Across all conditions, three distinct model/target viruses were rapidly inactivated to below the respective assay detection limit, even at Deviron 13‐S9 concentrations as low as 0.05% (v/v), that is, markedly below the lower limits of TX‐100 concentrations in current GMP processes. In addition, Vaccinia virus, considered as the worst‐case model with respect to detergent‐mediated clearance, was inactivated by Deviron 13‐S9 with similar kinetics as by a conventional, TX‐100‐containing S/D mix. Collectively, these results establish equivalent potency for virus inactivation, at a favorable use of only one detergent versus traditional S/D mixes from a biotechnological efficiency perspective.

## 1 Introduction

The lifesaving benefits of plasma‐derived medicines, for example, for treatment of blood clotting disorders or immunodeficiencies (Strengers 2017), were, in the 1980s, unfortunately associated with the tragedy of virus transmission to drug recipients. Particularly, plasma of donors infected with human immunodeficiency virus (HIV) and hepatitis C virus (HCV) resulted in contaminated plasma‐derived drugs that in turn infected drug recipients (Fletcher et al. 1983; Remis et al. 1990). To minimize any chance of reoccurrence, the “safety tripod” concept has been implemented, comprising (a) stringent qualification criteria for plasma donors, (b) serological and nucleic acid‐based testing of plasma with respect to certain viruses of concern, and (c) the addition of dedicated virus clearance steps, such as nanofiltration or solvent and detergent (S/D) treatment, in drug manufacture (Kreil 2018)., In terms of the risk reduction potential, it must be noted that the latter component constitutes the quantitatively most important factor of this strategy (Kreil 2018). Moreover, dedicated virus clearance steps, as integral parts of contemporary biopharmaceutical manufacturing processes, also provide safety measures against unknown or emerging viruses, as demonstrated in the past for West Nile virus (WNV) (Kreil et al. 2003; Poelsler et al. 2008), Chikungunya virus (CHIKV) (Leydold et al. 2012), and monkeypox virus (Kindermann et al. 2022).

As for S/D treatment of process intermediates, these compounds were revealed to destroy, at high efficiency, the lipid envelope of respective viruses (including, but not limited to, HIV or HCV), thereby rendering these potentially present contaminants irreversibly non‐infectious (Horowitz et al. 1985; 1992). The use of S/D mixes such as Triton X‐100 (TX‐100), tri‐n‐butyl phosphate (TnBP), and polysorbate 80 (PS80) for virus inactivation was subsequently also expanded to cell‐based biotechnological manufacturing processes, and indeed, no transmission events of enveloped viruses have been described for S/D‐treated products (Dichtelmüeller et al. 2009).

However, detrimental endocrine properties of TX‐100 degradation products (White et al. 1994), released, for example, from wastewater treatment plants, have resulted in the decision of European regulators to restrict and ultimately prohibit its use in the near future (ECHA n.d.). As a consequence, the pharmaceutical industry has started to identify surrogate compounds (Conley et al. 2017; Farcet et al. 2019; Hunter et al. 2022). Besides environmental sustainability and the ability to attack lipid envelopes without harming active pharmaceutical ingredients, such alternatives preferably should have similar chemical features as TX‐100, for example, a favorable viscosity and non‐ionic nature to support an implementation without the need for substantial process changes, and commensurate or even competitive pricing.

Recently, a secondary alcohol ethoxylate has been introduced as one such potential TX‐100 replacement (Banerjee et al. 2025; Yadav et al. 2025). With regard to its antiviral properties, this novel detergent—termed “Deviron 13‐S9” (hereinafter referred to as “Deviron”)—was shown to inactivate several enveloped model viruses at efficiencies that were comparable to TX‐100. The investigated matrices also included plasma (“cryo‐poor,” i.e., after removal of blood clotting factors), where Deviron or TX‐100 was combined with TnBP to generate a two‐component S/D mix.

The present study aimed at extending the comparative characterization of Deviron as a single detergent and established TX‐100‐based S/D combinations for use with plasma‐derived matrices, which occur further downstream in the manufacturing process of different drugs, that is, directly before the respective S/D treatment step. The chosen matrices are characterized by considerable differences in protein concentration and pH. Also, the robustness of virus inactivation was probed in further detail by applying, in these matrices, Deviron as a single detergent, also at markedly reduced concentrations compared to manufacturing, and by studying inactivation of *Vaccinia* virus (VACV), a well‐accepted “worst‐case” model with respect to virus inactivation (Roberts 2000).

## 2 Methods

### 2.1 Detergents

The following commercially available chemicals for S/D or detergent treatment were used as received from the vendors: Triton X‐100 (Merck), Polysorbate 80 (Brenntag CEE, GmbH), TnBP (Merck), Deviron (Merck, Millipore, article number 108694).

### 2.2 Process Intermediates

Process intermediates were obtained from large‐scale manufacturing of four plasma‐derived proteins: Immunoglobulins (IG), Fibrin, Thrombin, and coagulation factor VIII/von Willebrand factor complex (F VIII). All starting materials were drawn at the respective production stage prior to virus inactivation with S/D mix.

### 2.3 Virus Propagation, Cell Culture, and Virus Titration

Information on used viruses and cell lines is provided in Table 1. The panel of selected viruses corresponds to requirements defined in the relevant guideline (EMA 2011): human immunodeficiency virus (HIV) was chosen due to the documented HIV contamination of coagulation factor concentrates, bovine viral diarrhea virus (BVDV) as a model for hepatitis C virus (HCV), which has been transmitted via plasma derived medicines before the introduction of S/D treatment and Pseudorabies virus (PRV) as a model for the family *Herpesviridae*. In addition, VACV was included in the study design as a worst‐case model virus for detergent‐based treatment.

**Table 1:** Viruses and cell lines used for virus inactivation studies.

Virus	Strain (source)	Cell line for virus propagation (source)	Cell line for virus titration (source)
HIV	HIV‐1 IIIB (NIAID #398)	H9 (ECACC, 85050301)	AA2 (NIAID, #135)
BVDV	Nadl (ATCC VR‐1422)	MDBK (ATCC, CCL‐22)	BT (ATCC, CRL‐1390)
PRV	Kaplan (Eberhard Karls University, Tübingen, Germany)	Vero (ECACC, 84113001)	Vero (ECACC, 84113001)
Vaccinia	Bioreliance‐Acambis, Order number P/N IT‐0017, Lot I092701A	Vero (ECACC, 84113001)	Vero (ECACC, 84113001)

Abbreviations: AA2, lung epithelial cells; ATCC, American Type Culture Collection; BT, bovine turbinate tissue cells; BVDV, bovine viral diarrhea virus; ECACC, European Collection of Authenticated Cell Cultures; H9, T lymphocyte cells; HIV, human immunodeficiency virus; MDBK, Madin–Darby bovine kidney cells; NIAID, National Institute of Allergy and Infectious Diseases; PRV, pseudorabies virus; Vero, African green monkey kidney cells.

For H9 cells and AA2 cells, the growth medium consisted of RPMI1640 basal medium supplemented with 10% fetal calf serum (FCS), 2 mM l‐glutamine, 0.1 mg/mL gentamicin sulfate, 1 mM sodium pyruvate, and 1× nonessential amino acids. MDBK cells were cultivated in DMEM supplemented with 10% FCS, 2 mM l‐glutamine, 0.1 mg/mL gentamicin sulfate, 0.15% sodium bicarbonate, and 1× nonessential amino acids. For BT cells, the same medium was used except that FCS was replaced by 10% horse serum. For Vero cells, an in‐house proprietary basal medium was supplemented with 5% FCS, 2 mM l‐glutamine, 1 mM sodium pyruvate, 0.75% sodium bicarbonate, 0.1 mg/mL gentamicin sulfate, and 1× nonessential amino acids. All cell lines were cultivated under standard cell culture conditions (humidified incubators at 36°C and 5% CO_2_) and propagated via serial passaging employing trypsin for cell detachment.

For the determination of virus infectivity, median tissue culture infectious dose (TCID_50_) assays were employed. Briefly, from virus‐containing samples, a serial half‐log_10_ dilution series was established, which was applied in eightfold replicates per dilution onto 96‐well microtiter plates seeded with the respective indicator cell line. After incubation under standard cell culture conditions (humidified incubators at 36°C and 5% CO_2_), cytopathic effects were evaluated via bright‐field microscopy. TCID_50_ titers were calculated according to the Poisson distribution and expressed as log_10_[TCID_50_/mL]. Virus reduction factors (RFs) were calculated in accordance with the EU Committee for Proprietary Medicinal Products guidance (CPMP 1996). For samples after S/D or Deviron treatment, if no viral infectivity was detected in successive samples up until the final sample, the volume of all successive negative samples was taken into account for calculation of the assay detection limit and the resulting virus RF. The assay detection limit was dependent on the total volume of analyzed process intermediate (S/D or Deviron‐treated), as well as on the respective indicator cell line (different sample pre‐dilutions to bypass cytotoxic effects), and was in the range of 1.4–3.6 log_10_[TCID_50_/mL].

### 2.4 Virus Inactivation by Three‐Component S/D Treatment and Deviron

For all process intermediates, virus inactivation runs were conducted at a volume of approximately 30 mL under continuous mixing by a magnetic stirrer.

The intermediate derived from IG manufacture had a pH of 5.2 and was kept at 17°C ± 1°C. Protein concentration of the intermediate was determined by measuring absorbance in triplicate at 280–320 nm using a photometer (1 cm pathlength) and applying the Lambert–Beer law. To realize low, medium and high protein concentrations for studying virus inactivation, the intermediate was diluted to 7.2 g/L (runs with Deviron), 10.1 g/L (runs with the three‐component S/D mix containing Triton X‐100), 20.1 g/L (VACV inactivation runs with Deviron) or 50.2 g/L using a sodium chloride buffer (distilled water with 3 M NaCl added until a conductivity of 3.5 mS/cm was reached).

The intermediate derived from Fibrin manufacture had a pH of approximately 7.4 and a protein concentration of 27.3 g/L, while the intermediate derived from Thrombin manufacture had a pH of 7.3 to 7.5 and a protein concentration of 4.8 g/L. Virus inactivation runs with both intermediates were conducted at 19°C ± 1°C. The intermediate derived from coagulation factor VIII/von Willebrand factor complex had a pH of 7.3–7.6, a protein concentration of 7.3 g/L, and was kept at 23°C ± 1°C.

For HIV, BVDV, and PRV, spiking of virus stock solutions into process intermediates was performed at a ratio of 1:11, and two samples—spike control (SC) and hold control (HC)—were drawn, of which SC was titrated immediately, while HC was incubated in the same cooling unit as the detergent‐treated process material and titrated at the end of the experiment. For all manufacturing processes, the final target concentrations of PS80, TNBP, and TX‐100 are approximately 0.3%, 0.3%, and 1% (w/w), respectively. For each experimental run, the weight of the process material was determined to calculate the amount of TX‐100 (as part of the three‐component (S/D) mixture), or Deviron required to reach the respective final concentration, as summarized in Table 2. S/D mix or Deviron was added with either a Hamilton syringe or single‐use syringes, and the actual added amount was determined by backweighing the syringe. For IG, Fibrin, and F VIII intermediates, antiviral reagents were added after the virus spike, while for the Thrombin intermediate, Deviron or S/D mix was added first, followed by the virus spike. Samples for TCID_50_ virus titration were drawn at the following time points after S/D mix or Deviron addition: 30 s to 2 min, 10 ± 1 min, 15 ± 1 min (only for Thrombin intermediate and three‐component S/D mix), 20 ± 1 min, 30 ± 1 min, 59 ± 1 min, 69 ± 1 min (only for F VIII intermediate and three‐component S/D mix).

**Table 2:** Overview of virus inactivation runs.

	HIV/BVDV/PRV	VACV
	Spiking ratio [virus stock]:[process intermediate]	
	1:11	1:31
Process intermediate	Conc. of Deviron (single detergent) or TX‐100 (in S/D mix)	
IG	0.48% [v/v]	0.50% [v/v]
	0.05% [v/v]	0.10% [v/v]
Fibrin	0.68% [w/v]	n.d.
	0.05% [w/v]	
Thrombin	0.68% [w/w]	n.d.
	0.10% [w/w]	
F VIII	0.68% [w/v]	n.d.

*Note:* Per virus and process intermediate, chosen spiking ratios as well as final concentrations of Deviron (applied as single detergent) and TX‐100 (applied in combination with TNBP and PS80) are shown.

Abbreviations: BVDV, bovine viral diarrhea virus; HIV, human immunodeficiency virus; n.d., not done; PRV, pseudorabies virus; VACV, vaccinia virus.

Experiments employing VACV were conducted similarly, except that spiking of virus stock solutions into the IG process intermediate was performed at a ratio of 1:31.

For all used process materials, the cytotoxicity of the three‐component S/D mix or Deviron, as well as any possible matrix effects on cell lines used for virus detection, was tested and taken into consideration for the calculation of the RF.

## 3 Results

### 3.1 Virus Inactivation at the Lower Limit of Detergent Concentrations Employed in Manufacturing

**Figure 1:**
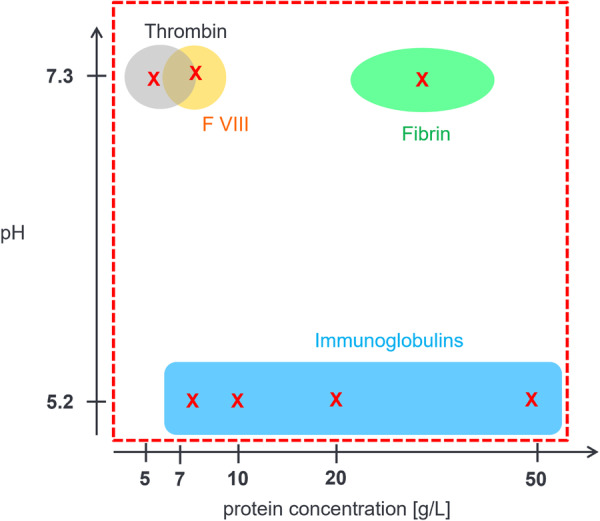
Virus inactivation study design space. Intermediates from manufacturing processes of four plasma‐derived products—Immunoglobulins (IG), Fibrin, Thrombin, and coagulation factor VIII/von Willebrand factor complex (FVIII)—were chosen to cover a broad range of pH values and protein concentration for investigation of virus inactivation due to Deviron in comparison to the established three‐component solvent/detergent (S/D) mix (TX‐100/TnBP/PS80). Actual conditions investigated are marked by red “X”.

To perform a holistic assessment of Deviron as a potential alternative to the established 3‐component S/D mix containing TX‐100, down‐scale virus inactivation studies were conducted using process intermediates from the manufacture of four different plasma‐derived products, which together cover a wide range of protein concentrations and pH values (Figure 1). In a first series of experiments, the concentration of Deviron or S/D mix was deliberately set close to, but slightly (i.e., 2%) below the lower acceptance limit defined for the respective large‐scale manufacturing process. Regardless of the investigated process intermediate, the target virus HIV was rapidly inactivated to below the detection limit, with similar or even greater RFs for Deviron used as single detergent, compared to the classical S/D mix (Table 3). Likewise, immediate and highly efficient inactivation of BVDV, a model virus for HCV, was observed across all matrices, with RF differences between Deviron and classical S/D mix always below 1 log_10_, that is, below the limit of assay reliability, as defined by applicable guideline (Table 3) (CPMP 1996). Also, PRV, a model for large enveloped DNA viruses, was efficiently inactivated to below the assay detection limit in all process intermediates, with largely comparable RFs for Deviron and S/D mix (Table 3). As seen from experiments with the IG process intermediate, where runs were conducted with samples spanning a fivefold difference in protein concentration, inactivation of all three viruses was largely comparable between different protein contents of the matrices.

**Table 3:** Inactivation of HIV, BVDV, and PRV by Deviron or three‐component S/D mix (TX‐100/TnBP/PS80), applied at final concentrations slightly below the lower manufacturing acceptance limit, in process intermediates derived from the manufacture of different plasma‐derived products.

	HIV		BVDV		PRV	
	Deviron	S/D mix	Deviron	S/D mix	Deviron	S/D mix
Process intermediate	RF/time until inactivation to below assay LoD					
IG high protein (50.2 g/L)	> 5.9/1 min	> 4.1/1 min	> 5.5/1 min	> 6.2/1 min	> 5.6/1 min	> 4.7/1 min
IG low protein (7.2 g/L with Deviron; 10.1 g/L with S/D mix)	> 6.0/1 min	> 4.8/1 min	> 5.9/1 min	> 6.1/1 min	> 5.3/1 min	> 5.5/1 min
Fibrin (27.3 g/L)	> 6.1/1 min	> 4.7/1 min	> 5.3/1 min	> 5.3/1 min	> 5.6/1 min	> 5.5/1 min
Thrombin (4.8 g/L)	> 5.6/10 min	> 5.3/1 min	> 5.7/1 min	> 5.3/1 min	> 5.3/1 min	> 6.2/1 min
F VIII (7.3 g/L)	> 5.3/1 min	> 4.7/1 min	> 5.7/1 min	> 5.2/10 min	> 5.5/1 min	> 6.0/10 min

*Note:* Data are shown as log_10_ reduction factor (RF), taking into account the volumes of successively drawn samples for which virus infectivity was below the assay limit of detection (LoD). For Fibrin and Thrombin intermediates, the RFs are the means of duplicate runs. For F VIII, for BVDV and PRV runs conducted with three‐component S/D mix, 10 min post S/D mix addition was the first kinetic timepoint assayed, and RFs are the mean of duplicate runs.

### 3.2 Virus Inactivation at Substantially Decreased Detergent Concentrations

**Figure 2:**
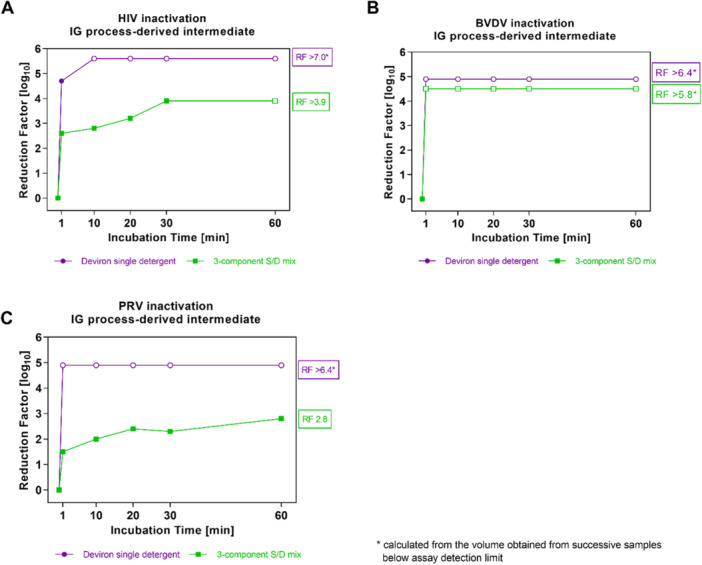
Kinetics of virus inactivation in Immunoglobulin (IG) process‐derived intermediate. The matrix (20.1 g protein/L; pH 5.2) was spiked with (A) human immunodeficiency virus (HIV), (B) bovine viral diarrhea virus (BVDV), or (C) pseudorabies virus (PRV) before addition of either TX‐100 (0.05% [v/v]) as part of the three‐component solvent/detergent (S/D) mixture, or Deviron (0.05% [v/v]). Samples were drawn after 1, 10, 20, 30, and 60 min for virus infectivity determination (TCID_50_ assay), and viral loads were compared to a sample drawn before S/D mix or Deviron addition to calculate the respective reduction factor (RF). Empty symbols indicate viral inactivation below the detection limit. Numerical RFs at the end of incubation are shown to the right; for runs with successive samples with undetectable virus infectivity, sample volumes were included for RF calculation.

To further characterize the effectiveness of Deviron‐based virus inactivation, a second series of down‐scale runs was conducted with drastically reduced detergent or S/D mix concentrations, that is, only at a final concentration of 0.1% or 0.05% (Table 2). These experimental designs should permit the study of inactivation kinetics, a regulatory requirement (CPMP 1996). Indeed, for IG process‐derived intermediate, while HIV clearance by the traditional S/D mix (applied at a concentration of 0.05%) was still rapid, inactivation to the assay detection limit was only reached at the last time point investigated (Figure 2). Incomplete, yet still robust inactivation was seen for PRV, while BVDV clearance was as rapid and effective as previously observed under higher S/D mix concentrations. The comparative single detergent runs revealed marked anti‐viral properties for Deviron: Rapid inactivation to below the detection limit was achieved already after only 10 min treatment time for the HIV spike experiments, while for all other conditions, clearance to below the assay detection limit was reached from the very first kinetic sample onwards, resulting in final RFs of > 7.0 log_10_ for HIV, > 6.4 log_10_ for BVDV, and > 6.4 log_10_ for PRV (Figure 2).

**Figure 3:**
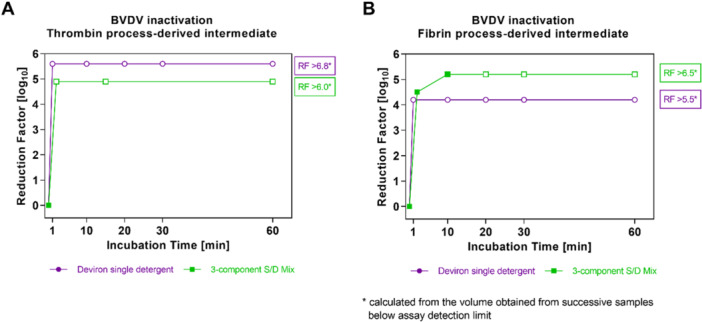
Kinetics of bovine viral diarrhea virus (BVDV) inactivation in Thrombin and Fibrin process‐derived intermediate. (A) To the Thrombin process‐derived matrix (4.8 g protein/L; pH 7.3), either TX‐100 (0.1% [w/w]) as part of the three‐component solvent/detergent (S/D) mixture, or Deviron (0.1% [w/w]) was added, followed by spiking with BVDV. Samples were drawn after 0.5, 10, 20, 30, and 59 min for the Deviron run, and after 1, 15, and 60 min for the run with S/D mix. (B) The Fibrin process‐derived matrix (27 g protein/L; pH 7.3) was spiked with BVDV before addition of either TX‐100 (0.05% [w/w]) as part of the three‐component S/D mixture, or Deviron (0.05% [w/w]). Samples were drawn after 1, 10, 20, 30, and 59 min. Virus infectivity in samples was determined by TCID_50_ assay, and viral loads were compared to a sample drawn before S/D mix or Deviron addition to calculate the respective reduction factor (RF). Empty symbols indicate viral inactivation below the detection limit. Numerical RFs at the end of incubation are shown to the right; for runs with successive samples with undetectable virus infectivity, sample volumes were included for RF calculation.

Comparable results were also obtained when focusing on a matrix of lower protein content, but higher pH, that is, process intermediate derived from the Thrombin manufacturing process: Again, BVDV was inactivated to below the assay detection limit already at the first kinetic sampling point, by both S/D mix and Deviron, ultimately yielding RFs of > 6.0 log_10_ and > 6.8 log_10_, respectively (Figure 3A). Finally, a similarly effective BVDV inactivation was determined in the process intermediate from the Fibrin manufacture (high protein content and high pH), that is, RFs of > 5.5 log_10_ for Deviron and > 6.5 log_10_ for S/D mix (Figure 3B).

Collectively, these series of experiments showed robust antiviral properties of Deviron used as single detergent, well comparable to those of the current three‐component S/D mix, even when applied at considerably lower final concentrations than specified for manufacturing of plasma‐derived pharmaceuticals.

### 3.3 Investigation of Detergent Performance Using a “Worst‐Case” Enveloped Virus

**Figure 4:**
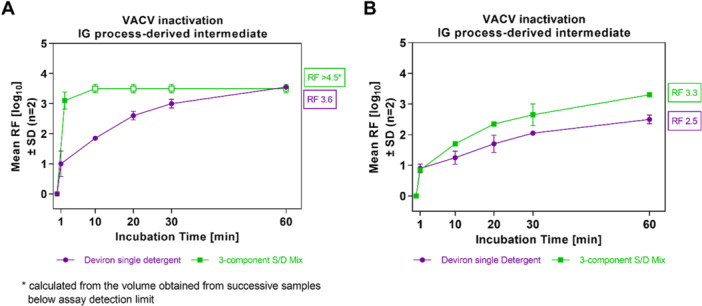
Kinetics of Vaccinia virus (VACV) inactivation in immunoglobulin (IG) process‐derived intermediate. (A) VACV was spiked into a matrix with high (50.1 g/L; Run 1) or low (10.1 g/L; Run 2) protein concentration before addition of TX‐100 (0.5% [w/v]) as part of the three‐component solvent/detergent (S/D) mixture. Deviron (0.5% [w/v]) was analyzed in two replicate runs with a matrix with medium (20.1 g/L) protein concentration. (B) VACV was spiked into a matrix with medium (20.1 g/L) protein concentration before the addition of either TX‐100 (0.1% [w/v]) as part of the three‐component S/D mixture, or Deviron (0.1% [w/v]), each analyzed in two replicate runs. Samples were drawn after 1, 10, 20, 30, and 60 min for virus infectivity determination (TCID_50_ assay), and viral loads were compared to a sample drawn before S/D mix or Deviron addition to calculate the respective reduction factor (RF). Error bars denote standard deviation (SD; only shown if larger than the height of symbols). Empty symbols indicate viral inactivation below the detection limit. Numerical RFs at the end of incubation are shown to the right; for runs with successive samples with undetectable virus infectivity, sample volumes were included for RF calculation.

VACV, a member of the *poxvirus* family, has been described as particularly resistant to S/D treatment (Remington et al. 2004; Roberts 2000), likely due to the existence of virion variants endowed with more than one lipid envelope layer (Smith et al. 2002). Inactivation of VACV thus represents a “worst‐case” when assessing the antiviral properties of a detergent. We therefore spiked the process intermediate from the IG manufacture with VACV to investigate the antiviral potency of Deviron and the S/D mix. At a final Deviron concentration of 0.47% [v/v], effective virus reduction in the order of 4 log_10_ was obtained with a VACV RF of 3.6 log_10_. The classical three‐component S/D mixture with TX‐100—at a final concentration of 0.50% [v/v]—evoked a faster VACV clearance to below the assay detection limit, resulting in a final RF of > 4.5 log_10_ (Figure 4A), which is still below the reliability threshold of 1 log_10_. As duplicate runs with a fivefold difference in protein concentration—independent of virus and detergent used—resulted in highly similar RFs (Table 2 and Figure 4A), the protein concentration was not varied at successive VACV inactivation runs with reduced detergent concentrations, that is, only 0.1% [v/v] of antiviral reagents. The corresponding experiments revealed somewhat decelerated virus inactivation kinetics with final RFs of 2.5 and 3.3 log_10_ for Deviron and the TX‐100‐containing S/D mix, respectively (Figure 4B).

## 4 Discussion

TX‐100 was identified during the 1980s HIV transmission crisis as a powerful antiviral nonionic detergent (Horowitz et al. 1985), and therefore has been widely established in routine GMP S/D treatment steps. However, indications of unfavorable environmental effects were subsequently discovered for its degradation products (Jobling et al. 1996; White et al. 1994). The ECHA eventually classified the superordinate substance group as “endocrine disrupting properties,” that is, octyl phenol ethoxylates were added to Annex XIV of the Registration, Evaluation, Authorization and Restriction of Chemicals (REACH) regulation framework (ECHA n.d.). While industries such as biopharma were given the possibility to use TX‐100 beyond the “sunset date” (January 2021), the corresponding authorization allows for continued use only for a limited time.

Following other TX‐100 replacement candidates (Chen et al. 2019; Conley et al. 2017; Farcet et al. 2019), Deviron has recently emerged from a systematic evaluation of several detergents, which was based on four criteria (solubility, critical micelle concentration [CMC], storage conditions, virus inactivation) (Yadav et al. 2025). Focusing on recombinant medicinal products, this study investigated the inactivation of PRV and X‐linked Murine Leukemia Virus (X‐MuLV; a model for retroviruses) by Deviron upstream, that is, in harvested cell culture fluid representative of a monoclonal antibody production process. At a detergent concentration of 0.3% (w/w; 25‐fold above the CMC), inactivation to the respective assay detection limit was observed within 10 min of treatment for both viruses. In a subsequent study, the characterization of Deviron was extended to more downstream matrices, which are also relevant for plasma‐derived pharmaceuticals (Banerjee et al. 2025). For cryo‐poor plasma, mixes of Deviron or TX‐100 with TnBP were able to inactivate PRV, X‐MuLV, and BVDV spikes to the respective assay detection limit, reaching RFs > 5 log_10_. In addition, this study also presented evidence of satisfactory detergent metabolization by microorganisms present in sewage sludge (i.e., data allowing to classify Deviron as “readily biodegradable”), as well as efficient detergent removal from the process stream via Protein A chromatography (Banerjee et al. 2025).

The present investigation aimed to characterize in more detail the suitability of Deviron for virus inactivation steps in the manufacture of plasma‐derived products. By extending our analyses to process intermediates from four distinct manufacturing processes, our study covers an unprecedentedly wide range of total protein concentration (from 5 to 50 g/L) and pH values (from 5.2 to 7.6). Also, compared to previous studies examining Deviron (Banerjee et al. 2025; Yadav et al. 2025), the final detergent concentration was further reduced by at least fivefold. The overall data for viruses of concern, including HIV (relevant due to its history of transmission events), revealed Deviron to have similarly potent viral inactivation properties as TX‐100. The robustness of such a single‐detergent treatment was corroborated as, even at concentrations markedly below what is used in manufacturing, all viruses were cleared to below the respective assay detection limit, for all process intermediates. Thereby, our study is also a confirmation of a previous S/D treatment meta‐analysis, which showed that neither matrix pH nor protein concentration has a significant impact on virus inactivation (Dichtelmueller et al. 2009).

As an important novelty, our study focused on the use of Deviron as single detergent for virus inactivation in plasma‐derived process intermediates. Even inactivation of the “worst‐case” VACV was reliable as compared to the traditional, TX‐100‐containing S/D mix. In line with previous single‐detergent studies with other matrices, the observed effective virus inactivation supports the establishment of corresponding large‐scale processes without TnBP; that is, future virus inactivation steps could be “detergent only” instead of S/D treatment. Besides the simplification of analytics, process monitoring, and compound removal procedures, this would also be favorable from a health and safety perspective, as TnBP is a CMR substance.

In summary, the present study establishes an encouraging basis for the use of the novel detergent Deviron as a single detergent across essentially all manufacturing processes of plasma‐derived medicines. Given the availability of Deviron at GMP‐grade, regulatory interactions for approval of process changes might be considered feasible, ultimately facilitating transition from TX‐100‐containing virus inactivation steps to improved, that is, environmentally compatible, alternatives in the near future.

## Author Contributions

Experimental conception and design of the study were performed by J.K., C.K., and T.R.K. Analysis and interpretation of data were carried out by C.K., J.K., and M.K. Draft manuscript was prepared by M.K., C.K., and J.K. All authors read and approved the final manuscript.

## Conflicts of Interest

All authors are employees of Takeda Manufacturing Austria AG. M.K. and T.R.K. have Takeda stock interest.

## Data Availability

The data that support the findings of this study are available from the corresponding author upon reasonable request.
